# Regulation of Proliferation, Differentiation and Functions of Osteoblasts by Runx2

**DOI:** 10.3390/ijms20071694

**Published:** 2019-04-04

**Authors:** Toshihisa Komori

**Affiliations:** Basic and Translational Research Center for Hard Tissue Disease, Nagasaki University Graduate School of Biomedical Sciences, Nagasaki 852-8588, Japan; komorit@nagasaki-u.ac.jp; Tel.: +81-95-819-7630; Fax: +81-95-819-7633

**Keywords:** Runx2, hedgehog, Wnt, Fgfr, Pthr1, Sp7, proliferation, differentiation, cleidocranial dysplasia, osteoblast

## Abstract

Runx2 is essential for osteoblast differentiation and chondrocyte maturation. During osteoblast differentiation, Runx2 is weakly expressed in uncommitted mesenchymal cells, and its expression is upregulated in preosteoblasts, reaches the maximal level in immature osteoblasts, and is down-regulated in mature osteoblasts. Runx2 enhances the proliferation of osteoblast progenitors by directly regulating *Fgfr2* and *Fgfr3*. Runx2 enhances the proliferation of suture mesenchymal cells and induces their commitment into osteoblast lineage cells through the direct regulation of hedgehog (*Ihh*, *Gli1*, and *Ptch1*), Fgf (*Fgfr2* and *Fgfr3*), Wnt (*Tcf7*, *Wnt10b*, and *Wnt1*), and Pthlh (*Pthr1*) signaling pathway genes, and *Dlx5*. *Runx2* heterozygous mutation causes open fontanelle and sutures because more than half of the *Runx2* gene dosage is required for the induction of these genes in suture mesenchymal cells. Runx2 regulates the proliferation of osteoblast progenitors and their differentiation into osteoblasts via reciprocal regulation with hedgehog, Fgf, Wnt, and Pthlh signaling molecules, and transcription factors, including Dlx5 and Sp7. Runx2 induces the expression of major bone matrix protein genes, including *Col1a1*, *Spp1*, *Ibsp*, *Bglap2*, and *Fn1*, in vitro. However, the functions of Runx2 in differentiated osteoblasts in the expression of these genes in vivo require further investigation.

## 1. Introduction

Runx2 belongs to the Runx family, which has the DNA-binding domain runt, and consists of Runx1, Runx2, and Runx3 [1]. Runx2 heterodimerizes with Cbfb and acquires enhanced DNA binding ability and protein stability [2,3,4,5,6]. *Runx2* has two promoters, P1 and P2, and the transcript from P1 encodes type II Runx2 and that from P2 encodes type I Runx2. Runx2 is expressed in osteoblasts and chondrocytes. In chondrocytes, Runx2 is weakly expressed in resting chondrocytes and upregulated in prehypertrophic chondrocytes, and this upregulated expression is maintained until terminal hypertrophic chondrocytes [7,8]. *Runx2*-deficient (*Runx2*^–/–^) mice lack osteoblasts and bone formation, and chondrocyte maturation is markedly inhibited [7,9,10,11]. *Col2a1*, which is expressed in immature chondrocytes, is expressed in whole *Runx2*^–/–^ skeletons, whereas *Col10a1*, which is expressed in hypertrophic chondrocytes, is restricted to the tibia, fibula, radius, and ulna. Runx3 is also expressed in chondrocytes, and its expression is upregulated in prehypertrophic chondrocytes and down-regulated in terminal hypertrophic chondrocytes. *Runx2*^–/–^*Runx3*^–/–^ mice lack hypertrophic chondrocytes, suggesting that Runx2 and Runx3 have redundant functions in chondrocyte maturation [12]. Runx2 is expressed in uncommitted mesenchymal cells, and its expression is upregulated in preosteoblasts, reaches the maximum level in immature osteoblasts, and is down-regulated in mature osteoblasts [13,14]. Furthermore, Runx2 regulates the proliferation of osteoblast progenitors, their commitment to osteoblast lineage cells, and the expression of bone matrix protein genes.

## 2. Reciprocal Regulation of the Essential Transcription Factors for Osteoblast Differentiation

**Figure 1:**
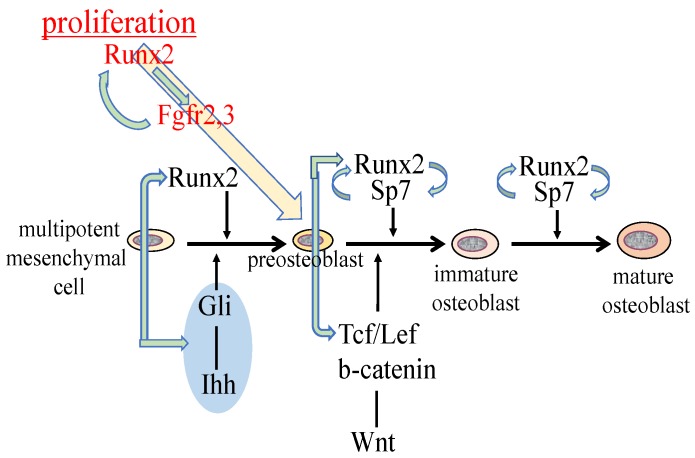
Regulation of osteoblast proliferation and differentiation by transcription factors. Runx2 induces the differentiation of multipotent mesenchymal cells into preosteoblasts. Ihh is required for the expression of *Runx2* in the perichondrium of endochondral bones. Runx2 induces *Sp7* expression, and Runx2, Sp7, and canonical Wnt signaling induce the differentiation of preosteoblasts into immature osteoblasts. Runx2 and Sp7 are also involved in the maturation of osteoblasts. Runx2 regulates the proliferation of preosteoblasts by inducing *Fgfr2* and *Fgfr3*. *Runx2* expression and that of hedgehog, Fgf, and Wnt signaling pathway genes, and *Sp7* are reciprocally regulated.

Hedgehog signaling is essential for osteoblast differentiation in endochondral bone. Hedgehog binding to Ptch relieves the repression of Smo, which ultimately regulates Gli [15]. Osteoblasts are absent in *Ihh*^–/–^ endochondral skeletons and *Runx2* expression is absent in the perichondrium [16]. *Ihh* conditional knockout mice created by crossing with *Col2a1* promoter Cre transgenic mice in which Cre is expressed in chondrocytes and perichondrial cells, which are progenitors of osteoblasts, do not form bone. Further, osteoblast marker gene expression, including *Runx2,* is absent from the perichondrium [17]. In *Smo* conditional knockout mice created by crossing with *Col2a1* Cre transgenic mice, *Runx2* expression is only induced outside of the perichondrium where osteoblast progenitors are located, and primary spongiosa is not formed, suggesting that hedgehog signaling is required for *Runx2* expression in the perichondrium and *Runx2*-expressing perichondrial cells are required for primary spongiosa formation [18] (Figure 1). However, the requirement of hedgehog signaling in osteoblast differentiation is transient because the deletion of *Smo* using *Sp7* promoter Cre transgenic mice in which Cre expression begins at the preosteoblast stage does not affect osteoblast differentiation [19]. The sources of osteoblasts in primary spongiosa have been demonstrated to be *Sp7*-expressing perichondrial cells and the transdifferentiated osteoblasts from hypertrophic chondrocytes [20,21,22]. Runx2 directly regulates *Ihh* expression in chondrocytes, osteoblast progenitors, and osteoblasts, as well as *Gli1* and *Ptch1* expression in osteoblast progenitors and osteoblasts [12,14]. Thus, Runx2 and hedgehog signaling regulate each other, and induce osteoblast differentiation (Figure 1).

Runx2 directly regulates *Sp7* expression, and osteoblasts and bone formation are also absent in *Sp7*^–/–^ mice [23,24]. As Runx2 is an upstream transcription factor of Sp7, Runx2 is expressed in *Sp7*^–/–^ mice [24,25]. One major difference between *Runx2*^–/–^ mice and *Sp7*^–/–^ mice is the number of mesenchymal cells in the presumptive bone regions. There are abundant mesenchymal cells that express Runx2 in the presumptive bone regions in *Sp7*^–/–^ mice, whereas there are few in these regions in *Runx2*^–/–^ mice [25]. This suggests that Runx2 is required for the expansion of mesenchymal cells, as described in detail later. As Sp7 activates an osteoblast-specific enhancer of *Runx2*, Sp7 is also involved in the regulation of *Runx2* expression [26] (Figure 1). Canonical Wnt signaling is also essential for osteoblast differentiation. In conditional *Ctnnb1* knockout mice created by crossing with *Twist2* (*Dermo1*) Cre knock-in mice in which *Ctnnb1* is deleted in osteo-chondroprogenitors, osteoblasts are absent, *Runx2* is expressed in the perichondrial cells, and *Sp7* expression is weak or absent in perichondrial cells [27,28]. Conditional *Ctnnb1* knockout mice created by crossing with *Col2a1* promoter Cre transgenic mice or *Prrx1* promoter Cre transgenic mice in which *Ctnnb1* is deleted in osteoblast progenitors in calvaria and osteo-chondroprogenitors in limb skeletons also lack osteoblasts in the Cre-expressing skeletons [19,29]. Similar to *Sp7*^–/–^ mice, conditional *Ctnnb1*^–/–^ mice also have abundant mesenchymal cells that express Runx2 in the presumptive bone region, suggesting again that Runx2 is required for the expansion of mesenchymal cells. As some of the mesenchymal cells in the perichondrium and calvaria differentiate into chondrocytes in *Sp7*^–/–^ mice and conditional *Ctnnb1*^–/–^ mice, Sp7 and canonical Wnt signaling inhibit chondrocyte differentiation and direct Runx2^+^ osteoblast progenitors to become osteoblasts (Figure 1).

## 3. Regulation of the Proliferation of Osteoblast Progenitors by Runx2

Overexpression of *Runx2* under the control of the *Prrx1* promoter accelerated osteoblast differentiation, inhibited chondrocyte differentiation, and caused limb defects [30]. The epithelial-mesenchymal interaction loop formed by Fgfs and Fgfrs is essential for limb development. The epithelial-mesenchymal interaction is formed by the different affinities of Fgfs for Fgfr1-3 with the alternatively spliced Ig-like domains of IIIb (“b”) and IIIc (“c”) [31]. Fgf10, which is expressed in the mesenchyme of the limbs, has a high affinity for Fgfr2b in the apical ectodermal ridge (AER), and induces Fgf4 and Fgf8 in the AER. Fgf4 and Fgf8 have a high affinity for Fgfr1c and Fgfr2c, which are expressed in the mesenchyme, and induce the proliferation of the mesenchymal cells. The limb defects in *Runx2* transgenic mice were caused by the induction of IIIb isoforms of Fgfr1 and Fgfr2, which have a high affinity for Fgf10, in the mesenchyme, leading to failure of the AER formation and Fgf4 and Fgf8 induction in that region. Consistent with *Fgfr1-3* induction in *Runx2* transgenic mice, overexpression or knockdown experiments, and reporter and ChIP assays demonstrated that Runx2 directly regulates *Fgfr1*, *Fgfr2*, and *Fgfr3* [25].

Among Fgfr1-3, the expression levels were *Fgfr1*>*Fgfr2*>*Fgfr3* in calvarial tissues. Fgfr2 and Fgfr3 play roles in the proliferation of osteoblast progenitors, and they induce proliferation through the mitogen-activated protein kinases (MAPK) signaling pathway. The expression of *Fgfr1-3* was markedly reduced in *Runx2*^–/–^ calvaria but not in *Sp7*^–/–^ calvaria. Runx2 increased the proliferation of wild-type osteoblast progenitors and augmented Fgf2-induced proliferation. However, Wnt3a, Ihh, Shh, and PTHrP (1-34) failed to increase the proliferation or augment *Runx2*-induced proliferation. Thus, Fgf signaling plays a major role in the proliferation of osteoblast progenitors, and Runx2 regulates the proliferation of osteoblast progenitors by inducing *Fgfr2* and *Fgfr3* [25] (Figure 1).

However, in all of the previous reports, Runx2 negatively regulated the proliferation of osteoblast lineage cells. *Runx2*^–/–^ calvarial cells proliferated faster than wild-type calvarial cells in vitro [32]. *Runx2* expression was up-regulated in the G0 phase and down-regulated in the G1 phase of the cell cycle of MC3T3-E1 preosteoblastic cells [33]. Furthermore, Runx2 suppressed the proliferation of cells with osteogenic potential and osteosarcoma cells, and the introduction of siRNA against *Runx2* into human mesenchymal stem cells increased their proliferation [34,35]. Moreover, Runx2 induced G1 cell-cycle arrest through the induction of *Cdkn1b* (p27^KIP1^) in osteosarcoma cells, the expression of *Cdkn1a* (p21^CIP1^) and *Cdkn2a* (p19^ARF^) was reduced in *Runx2*^–/–^ osteoblast progenitors in vitro, and the introduction of *Runx2* induced the expression of *Cdkn1b*, *Cdkn1a*, and *Cdkn2a*, suggesting that they are targeting genes responsible for the inhibitory function of Runx2 in the proliferation of osteoblast lineage cells [36,37]. In the previous report, marked reduction of *Cdkn1a* and *Cdkn2a* expression in *Runx2*^–/–^ calvarial cells occurred after six passages of the cells [37]. In our analysis using *Runx2*^–/–^ calvarial cells after one passage, *Cdkn1b* expression in *Runx2*^–/–^ calvarial cells was greater than that in wild-type calvarial cells, *Cdkn1a*, *Cdkn2a* (p19^ARF^), and *Cdkn2a* (p16^Ink4a^) expression in *Runx2*^–/–^ calvarial cells was approximately 75% of that in wild-type calvarial cells, and the introduction of *Runx2* failed to induce the expression of these genes [25]. Moreover, the expression profiles of *Runx2*^–/–^ calvarial cells compared with wild-type calvarial cells differed between in vitro and in vivo samples, thereby explaining the discrepancies between the in vitro and in vivo findings [25].

It is difficult to investigate the proliferation of osteoblast lineage cells in vivo because osteoblast lineage cells at different stages of differentiation are mixed and the differentiation stage affects their proliferation. Both *Runx2*^–/–^ mice and *Sp7*^–/–^ mice have cartilaginous skeletons, and lack osteoblasts and bone formation [9,10,24]. *Sp7*^–/–^ mice have abundant mesenchymal cells, which express *Col1a1* weakly and are actively proliferating, in the presumptive bone regions, whereas *Runx2*^–/–^ mice have few mesenchymal cells, which express *Col1a1* at a markedly low level and have low proliferative activity, in the presumptive bone regions [25]. Furthermore, Runx2, Fgfr2, and Fgfr3 are expressed in the mesenchymal cells in *Sp7*^–/–^ mice at levels comparable to those in osteoblasts in wild-type mice. These suggest that mesenchymal cells in *Sp7*^–/–^ mice are preosteoblasts and that *Sp7*^–/–^ mice are an appropriate model for the investigation of preosteoblast proliferation because osteoblast differentiation is blocked at the preosteoblast stage. *Sp7*^–/–^ preosteoblasts proliferated at a similar level as wild-type osteoblast lineage cells in vivo, but they proliferated faster than wild-type osteoblast progenitors in vitro. Fgf2 augmented the proliferation of *Sp7*^–/–^ preosteoblasts, whereas knockdown of *Runx2* inhibited this augmentation and reduced the expression of *Fgfr2* and *Fgfr3*. The amount and proliferation of preosteoblasts in *Sp7*^–/–^ mice was halved in *Sp7*^–/–^*Runx2*^+/–^ mice, indicating that preosteoblast proliferation is dependent on the gene dosage of *Runx2*. Therefore, Runx2 is required for preosteoblast proliferation in vivo, and Runx2 regulates it through the induction of *Fgfr2* and *Fgfr3* [25]. Moreover, Fgf2 enhances the Runx2 capacity for transcriptional activation via the PKC and MAPK pathways [25,38,39,40,41]. Thus, the Fgf signaling pathway and Runx2 positively regulate each other (Figure 1).

## 4. Molecular Mechanism of the Pathogenesis of Open Fontanelles and Sutures in Cleidocranial Dysplasia (CCD)

Although both intramembranous and endochondral bone development are affected in cleidocranial dysplasia (CCD), the open fontanelles and sutures and hypoplastic clavicles are typical features of CCD [5,42,43]. However, why the development of calvaria and clavicles is the most severely affected in CCD remains unclear. The posterior frontal (PF) suture is unique among the processes of suture closure. Mesenchymal condensation occurs at the PF suture at around P7 in mice, when osteogenic fronts closely face each other, and the mesenchymal cells differentiate into chondrocytes. Chondrocytes mature, cartilage is replaced with bone through endochondral ossification, each side of the frontal bone completely fuses, and the PF suture completely closes radiologically and histologically [44,45]. The differentiation of suture mesenchymal cells into chondrocytes is likely caused, at least in part, by the reduction of Wnt signaling, which inhibits chondrocyte differentiation, in the PF suture when the osteogenic fronts face each other [14,46]. The process of endochondral ossification is not observed in the other sutures, including the sagittal (SAG) suture, and the sutures close radiologically, but both sides of calvarial bones do not fuse histologically.

**Figure 2:**
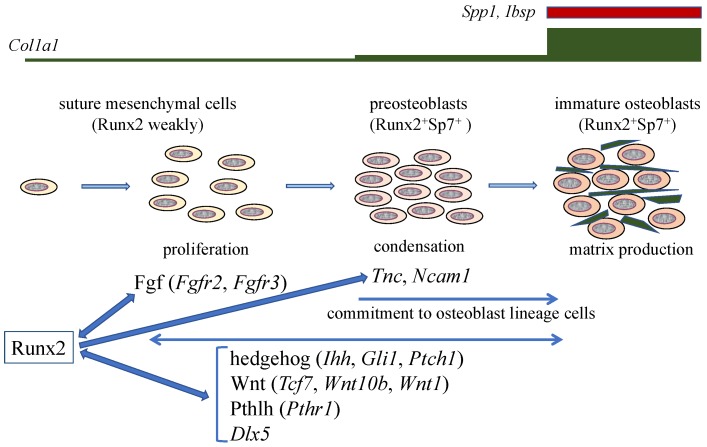
Calvarial bone development and suture closure. Suture mesenchymal cells weakly express *Runx2*, and its expression is upregulated in preosteoblasts and reaches the maximum level in immature osteoblasts. Runx2 induces *Sp7* expression at the preosteoblast stage. *Col1a1* expression is weak in suture mesenchymal cells, slightly upregulated in preosteoblasts, and markedly upregulated in immature osteoblasts, which also express *Spp1* and *Ibsp*. Runx2 increases the proliferation of suture mesenchymal cells and induces their commitment into osteoblast lineage cells through the induction of Fgf (*Fgfr2* and *Fgfr3*), hedgehog (*Ihh*, *Gli1*, and *Ptch1*), Wnt (*Tcf7*, *Wnt10b*, and *Wnt1*), and Pthlh (*Pthr1*) signaling pathway genes, and *Dlx5*. Fgf signaling plays a role in proliferation, whereas the other genes function in both proliferation and commitment. There is reciprocal regulation between Runx2, and these signaling pathways and Dlx5. In the processes of commitment into osteoblast lineage cells, Runx2 also induces *Tnc* and *Ncam1*, which likely play roles in the condensation of suture mesenchymal cells, and these condensed mesenchymal cells then become preosteoblasts. The preosteoblasts become immature osteoblasts in the sagittal (SAG) suture, but not in the posterior frontal (PF) suture, where they become chondrocytes due, at least in part, to the reduction of Wnt signaling.

In *Runx2*^+/–^ mice, the closure of both PF and SAG sutures was interrupted, and cartilaginous tissue was not observed in the PF suture. The suture mesenchymal cells expressed Sox9 at a similar level in wild-type and *Runx2*^+/–^ mice. The suture mesenchymal cells also expressed Runx2, but the expression level in *Runx2*^+/–^ mice was half of that in wild-type mice. The cell density and cell proliferation in *Runx2*^+/–^ sutures was less than those in wild-type mice. The expression of hedgehog signaling genes (*Gli1*, *Ptch1*, and *Ihh*), Fgf signaling genes (*Fgfr2 and Fgfr3*), Wnt signaling genes (*Tcf7 and Wnt10b*), *Pth1r, Dlx5, Tnc, and Ncam1* were less in PF and SAG sutures of *Runx2*^+/–^ mice than in those of wild-type mice. Overexpression or knockdown of *Runx2* and ChIP analysis demonstrated that these genes are directly regulated by Runx2 (Figure 2). However, the expression levels of these genes, except Dlx5, were similar in calvarial bone tissues between wild-type and *Runx2*^+/–^ mice. Furthermore, osteoblast marker gene expression was not reduced in the calvarial bone tissues of *Runx2*^+/–^ mice. These findings indicate that more than half of the *Runx2* gene dosage is required for the expression of these Runx2 target genes in suture mesenchymal cells, but half of the *Runx2* gene dosage is sufficient for it in differentiated osteoblasts [14].

In organ culture of *Runx2*^+/–^ calvaria, the ligands or agonists for hedgehog, Fgf, Wnt, and Pthlh signaling pathways enhanced calvarial bone development and suture closure. Furthermore, the antagonists of hedgehog, Fgf, Wnt, and Pthlh signaling pathways inhibited calvarial bone development, suture closure, and proliferation of suture mesenchymal cells in the organ culture of wild-type calvaria. These findings suggested that hedgehog, Fgf, Wnt, and Pthlh signaling pathways are involved in the expansion, condensation, and commitment of suture mesenchymal cells to osteoblast lineage cells, and that Runx2 regulates these processes by inducing their signaling pathway genes [14] (Figure 2).

Runx2 directly regulates the expression of *Tcf7*, *Wnt10b*, and *Wnt1*, and Tcf7 and Ctnnb1 activate the P1 promoter and osteoblast-specific enhancer of *Runx2* [14,26,47,48]. Thus, Wnt signaling and Runx2 mutually regulate their expression (Figure 1 and Figure 2). As Dlx5 activates the P1 promoter and osteoblast-specific enhancer of *Runx2* [26,49], Dlx5 and Runx2 also mutually regulate their expression (Figure 2). In addition, parathyroid hormone (PTH) increases *Runx2* mRNA, Runx2 protein, and Runx2 activity, PTH induces *Mmp13* promoter activity by activating Runx2 through PKA, and intermittent administration of PTH exerts lower anabolic effects on osteoblast-specific dominant-negative *Runx2* or overexpressing *Runx2* transgenic mice [13,50,51,52]. PTH and Pthlh share a common signaling pathway; therefore, there is also reciprocal regulation between the Pthlh signaling pathway and Runx2 (Figure 2).

## 5. The Functions of Runx2 in Bone Matrix Protein Gene Expression

After commitment to osteoblastic lineage cells, the osteoblasts express bone matrix protein genes at different levels depending on the maturational stage of the cells. Uncommitted mesenchymal cells weakly express *Col1a1*, its expression is slightly upregulated in preosteoblasts, and is markedly upregulated in immature osteoblasts [14] (Figure 2). Immature osteoblasts express *Spp1* and then *Ibsp*, and mature osteoblasts strongly express *Col1a1* and *Bglap2* [13,53] (Figure 2). Mature osteoblasts are embedded into the bone matrix and become immature osteocytes, which express *Dmp1*, and become mature osteocytes, which express *Sost* [54,55,56].

*Runx2*^–/–^ mice lack osteoblasts, and the expression of bone matrix protein genes, including *Spp1*, *Ibsp*, and *Bglap2*, is absent and *Col1a1* expression is very low in the presumptive bone regions [7,9]. Although osteoblasts are observed in type II *Runx2*-specific knockout mice, the expression of *Col1a1*, *Spp1*, and *Bglap2* is reduced [57]. In vitro studies also demonstrated that Runx2 is a positive regulator that can upregulate the expression of bone matrix protein genes, including *Col1a1*, *Spp1*, *Ibsp*, *Bglap2*, and *Fn1* [58,59,60,61]. Moreover, reporter assays revealed that Runx2 activates the promoters of bone matrix protein genes, including *Col1a1*, *Col1a2*, *Spp1*, and *Bglap2* [59,60,62,63,64].

The most important function of osteoblasts is the production of type I collagen, which composes 90% of bone. Type I collagen is a heterotrimeric protein comprising two α1(I) chains and one α2(I) chain, which are encoded by *Col1a1* and *Col1a2*, respectively. Reporter mice demonstrated that a 0.9-kb *Col1a1* promoter directs weak reporter gene expression in skin fibroblasts, a 2.3-kb *Col1a1* promoter directs additional strong reporter gene expression in osteoblasts and odontoblasts, and a 3.2-kb *Col1a1* promoter directs additional strong reporter gene expression in tendon and fascia fibroblasts [65]. Reporter mice covering the *Col1a2* gene locus clarified the presence of an enhancer in the region between −13.5 and −19.5 kb of the *Col1a2* gene [66].

In a reporter assay, a Runx2 binding motif, which is conserved in mice, rats, and humans, located at −1347 in the mouse *Col1a1* gene was found to be responsible for transcriptional activation by Runx2 [63]. However, deletion of the comparable region in rats had no effect [67]. Conditional deletion of the runt domain of *Runx2*, which is required for DNA binding, in osteoblasts using 2.3-kb *Col1a1* promoter Cre transgenic mice resulted in similar bone volume and bone formation as in wild-type mice [68]. In contrast, conditional deletion of exon 8 of *Runx2* using the same 2.3-kb *Col1a1* promoter Cre transgenic mouse line resulted in the expression of cryptic Runx2 protein, which has a lower capacity for transcriptional activation, leading to reduced bone mass [69]. Although the expression of *Col1a1* and *Col1a2* was not examined in either report, their expression is likely unaffected in the former and reduced in the latter. In the latter case, however, the cryptic Runx2 protein retains the capacity for nuclear import and DNA binding. Therefore, the cryptic Runx2 protein may reduce bone mass by inhibiting the binding of Runx3, because the deletion of *Runx3* in osteoblasts using the 2.3-kb *Col1a1* promoter Cre transgenic mice results in osteopenia [70]. Although *Col1a1* expression has been reported to precede *Runx2* expression [71], *Col1a1* expression in uncommitted mesenchymal cells of *Runx2*^–/–^ mice and preosteoblasts of *Sp7*^–/–^ mice is very weak, and *Col1a1* expression is greatly upregulated after commitment into osteoblasts in wild-type mice [14] (Figure 2). Therefore, the involvement of Runx2 in *Col1a1* expression in differentiated osteoblasts in vivo requires further investigation.

Sp7 is involved in *Col1a1* expression in vitro, and Runx2 and Sp7 cooperatively regulate *Col1a1* expression, which is augmented through phosphorylation by p38 and ERK [72,73,74]. The bone mass was reduced in vertebrae, trabecular bone was increased and cortical bone was reduced in femurs, and *Col1a1* expression was unchanged in *Sp7*^fl/–^;2.3-kb *Col1a1* Cre mice [75]. In *Sp7* ^fl/–^;2.3-kb *Col1a1* CreERT2 mice, the induction of Cre activity by 4-hydroxytamoxifen reduced bone mass in vertebrae and *Col1a1* expression in long bones [76]. Although the results are inconsistent, Sp7 may play a role in *Col1a1* expression in differentiated osteoblasts in vivo.

## 6. Conclusions

Runx2 is required for the proliferation of preosteoblasts in whole skeletons and mesenchymal cells in sutures. Indeed, Runx2 is required for the commitment of mesenchymal cells to osteoblast lineage cells. Thus, Runx2 makes a condensed cell layer of uncommitted mesenchymal cells or osteoblast progenitors by increasing their proliferation and facilitates their differentiation into osteoblast lineage cells. Runx2 can exert multiple functions through reciprocal regulation via major signaling pathways, including Fgf, hedgehog, Wnt, and Pthlh, and transcription factors, including Sp7 and Dlx5. Osteoblast proliferation and differentiation are likely regulated by such reciprocal regulation rather than the cascade of transcription factors.
